# NR4A1 Knockdown Suppresses Seizure Activity by Regulating Surface Expression of NR2B

**DOI:** 10.1038/srep37713

**Published:** 2016-11-23

**Authors:** Yanke Zhang, Guojun Chen, Baobing Gao, Yunlin Li, Shuli Liang, Xiaofei Wang, Xuefeng Wang, Binglin Zhu

**Affiliations:** 1 Department of Neurology, The First Affiliated Hospital of Chongqing Medical University, Chongqing Key Laboratory of Neurology, 1 Youyi Road, Chongqing 400016, China; 2 Department of Neurology, Chongqing General Hospital, 104 Pipashan Street, Chongqing 400010, China; 3 Department of Neurosurgery, The Affiliated Children’s Hospital of Capital Institute of Pediatrics, 2 Yabao Road, Beijing 100020, China; 4 Department of Neurosurgery, The First Affiliated Hospital of PLA General Hospital, 51 Fucheng Road, Beijing 100048, China; 5 Department of Neurology, The Affiliated Children’s Hospital, Capital Medical University, 56 Nanlishi Road, Xicheng District, Beijing 100032, China; 6 Center of Epilepsy, Beijing Institute for Brain Disorders, 10 Xitoutiao, Youanmen, Fengtai District, Beijing 100069, China

## Abstract

Nuclear receptor subfamily 4 group A member 1 (NR4A1), a downstream target of CREB that is a key regulator of epileptogenesis, has been implicated in a variety of biological processes and was previously identified as a seizure-associated molecule. However, the relationship between NR4A1 and epileptogenesis remains unclear. Here, we showed that NR4A1 protein was predominantly expressed in neurons and up-regulated in patients with epilepsy as well as pilocarpine-induced mouse epileptic models. NR4A1 knockdown by lentivirus transfection (lenti-shNR4A1) alleviated seizure severity and prolonged onset latency in mouse models. Moreover, reciprocal coimmunoprecipitation of NR4A1 and NR2B demonstrated their interaction. Furthermore, the expression of p-NR2B (Tyr1472) in epileptic mice and the expression of NR2B in the postsynaptic density (PSD) were significantly reduced in the lenti-shNR4A1 group, indicating that NR4A1 knockdown partly decreased surface NR2B by promoting NR2B internalization. These results are the first to indicate that the expression of NR4A1 in epileptic brain tissues may provide new insights into the molecular mechanisms underlying epilepsy.

Epilepsy, a common and devastating neurological disorder, is predominantly characterized by recurrent unprovoked epileptic seizures and affects approximately 50 million people worldwide^1^,^2^,^3^,^4^. Epileptic seizures are not adequately controlled by currently available antiepileptic drugs (AEDs), resulting in poor outcomes in some patients. Temporal lobe epilepsy (TLE), characterized by chronic spontaneous recurrent seizures (SRS), is the most common form of epilepsy, is often refractory to AEDs^5^ and involves dysregulation of amygdalo-hippocampal function caused by neuronal hyper-excitability^6^. Many signaling pathways are involved in seizure-induced cognitive dysfunction, long-term behavioral and neuronal apoptosis^7^,^8^. However, the molecular mechanisms underlying epilepsy are still unclear.

Nuclear receptor subfamily 4 group A member 1 (NR4A1), a member of the nuclear receptor family of transcription factors, belongs to the NR4A subfamily, which has been implicated in a variety of biological processes, such as cell apoptosis, proliferation, inflammation, and metabolism^9^. Notably, NR4A receptors are significantly induced in the CNS by stimuli such as seizures^10^,^11^ and focal brain injury^12^. NR4A1 has previously been identified as a seizure-associated molecule and is up-regulated following seizure induction in the hippocampus, including area CA1, using RNA transfer or *in situ* hybrization^10^,^13^,^14^. cAMP-response element-binding protein (CREB), a key regulator of epileptogenesis, is a transcription factor that is activated in response to stressful stimuli, such as hypoxia, oxidative stress, excitotoxicity, and ischemia^15^. CREB has been showed to play an important role in epilepsy. Immunostaining of activated CREB (Ser133-phosphorylated CREB, p-CREB) was significantly stronger in the hippocampus of mice with pilocarpine-induced seizure compared with control mice^16^,^17^,^18^. In the CNS, NR4A1 expression is controlled by N-methyl-D-aspartate receptors (NMDARs) and CREB, which are key regulators of synaptic function^19^,^20^. NR4A1 is a downstream target of the CREB^21^, and CREB has been reported to regulate NRA41 expression in various cell types^22^,^23^,^24^. Currently, the developmental and physiological functions of NR4A1 are poorly understood.

To date, the expression of NR4A1 in the brain tissues of epileptic patients has not been investigated. Whether the CREB/NR4A1 signaling pathway is involved in TLE pathogenesis is still unclear. We found that NR4A1 was up-regulated in epilepsy, and NR4A1 knockdown alleviated seizure severity. Furthermore, reciprocal coimmunoprecipitation showed that NR4A1 interacted with NR2B. The levels of p-NR2B (Tyr1472) in epileptic mice and the expression of NR2B in postsynaptic density (PSD) were significantly reduced in the NR4A1 knockdown group. Thus, our findings provide valuable information about the mechanisms of human TLE.

## Experimental procedures

### Human brain tissues

The study was performed with the formal consent of the patients or their lineal relatives for the use of data and brain tissues and approved by the Ethics Committee of Chongqing Medical University. Written informed consent was obtained from all patients or their lineal relatives. This study was conducted in accordance with the Declaration of Helsinki.

Neocortical tissue samples from twenty-four patients undergoing surgery for medically intractable epilepsy and ten control samples obtained from non-epileptic patients undergoing surgical therapy for post-trauma intracranial hypertension were randomly chosen from the brain tissue bank established in our lab, and the clinical data from the human brain tissue bank have been reported in our previous studies^25^,^26^. The diagnosis of epilepsy was confirmed in these patients according to criteria established by the International League Against Epilepsy^27^. Inclusion criteria were as follows: typical clinical manifestations; brain MRI or CT found no other neurological diseases; 24 h abnormal electroencephalogram (EEG); and pathological histological changes (Table 1). In the control cases, there was no history of epilepsy and exposure to AEDs and no other CNS diseases.

A part of the excised brain tissue sample from each TLE patient and control patient was stored in liquid nitrogen until used for Western blot analysis. The remaining part of the samples was fixed in 4% paraformaldehyde (PFA) for 24 h, embedded in paraffin, and sectioned at 10 μm for immunofluorescence analysis.

### Mouse models of epilepsy

All protocols were approved by the Commission of Chongqing Medical University for Ethics of Experiments on Animals and were conducted in accordance with international standards.

Forty-six healthy adult male C57 BL/6 mice (8 weeks old, 20–22 g) from the Experimental Animal Center of Chongqing Medical University were housed in a 12 h light/dark cycle with ad libitum access to food and water. The procedures conformed to the Commission of Chongqing Medical University for the Ethics of Experiments on Animals in accordance with international standards.

The pilocarpine epileptic mouse model was established as previously reported^28^. In brief, all mice (n = 46) underwent an intraperitoneal (i.p.) injection of 1 mg/kg methyl-scopolamine (Sigma, St. Louis, MO, USA) 30 min before pilocarpine administration to minimize the peripheral effects of cholinergic stimulation. Then, 100 mg/kg of pilocarpine was injected (i.p.) every 20 min until the onset of status epilepticus (SE). Behaviors were continuously observed, and the evoked SE was rated from 1 to 5 according to Racine’s scale^29^. Twenty-six mice (20 mice died from seizures) displaying stage 4 or 5 behaviors for 60 min were injected (i.p.) with 10 mg/kg diazepam to terminate SE. To establish the pilocarpine epilepsy model, the mice displaying spontaneous recurrent seizures (SRS) were regarded as the chronic phase pilocarpine epilepsy model (described as “epilepsy” in figures, n = 16). In comparison, the mice with no SRS were regarded as the control group (described as “control” in figures, n = 10). The electroencephalographic (EEG) activity was obtained in freely moving mice using a 16-channel electrophysiological data acquisition system (OmniPlex, Plexon, Dallas, TX, USA) as previously described in our laboratory^26^.

All mice (n = 26) were anesthetized by an injection (i.p.) of 3.5% chloral hydrate (1 ml/100 g). Eighteen mice (epilepsy, n = 12; control, n = 6) were decapitated, and the hippocampus and cortex were isolated and stored in liquid nitrogen for Western blot or immunoprecipitation analysis. The remaining mice (epilepsy, n = 4; control, n = 4) were intracardially perfused using 0.9% saline followed by 4% PFA, and the brains were immediately removed and postfixed in PFA for 24 h, embedded in paraffin, and sectioned at 10 μm for immunofluorescence analysis.

### Lentiviral vector injections

The recombinant lentiviral vector with mouse NR4A1 shRNA, along with the transgene for green fluorescent protein (GFP), which was driven by an Ubi promoter, was manufactured by Shanghai GeneChem Co., Ltd. (Shanghai, China). A universal scrambled sequence with mismatched bases was used as the negative control. The control shRNA (lenti-scr) targeting sequence is 5′-TTCTCCGAACGTGTCACGT-3′; the sequence of NR4A1 shRNA (lenti-shNR4A1) was 5′-TTTCTGTACTGTGCGCTTG-3′, which was previously showed to efficiently down-regulate mouse NR4A1 expression^30^. The shRNA target sequences were inserted into the GV118 lentivector between unique *Age*I and *EcoR*I sites. The GV118 lentivectors containing the shRNA sequences were transfected into 293 T cells, and viral supernatants were harvested after 48 h. The titers were determined with serial dilutions of concentrated lentivirus. The titer was 5 × 10^8^ TU/mL.

Mice were randomly divided into 2 groups: lenti-shNR4A1 group (n = 12) and lenti-scr group (n = 12). All mice were deeply anesthetized by intraperitoneal injections of 3.5% chloral hydrate (0.01 mL/g) and were positioned in a stereotaxia frame (Stoelting Co., Ltd., USA). The lentiviral particles (3 μL per side) were microinjected bilaterally into the dorsal hippocampus dentate gyrus region using a glass pipette (0.2 μL/min). The pipette was left in place for at least 5 min after injection to prevent backflow. Successful lentivirus infection could be detected after 5 days of injection. The lentivirus transfection lasted for the life span of the mice after successful inoculation into host cells. Two weeks after administration of lentiviral vectors, we induced seizures with pilocarpine as described above. Mice behavior was monitored continuously by a video surveillance system. We recorded 24 h/day for 21 consecutive days, beginning on the 14th day after SE. The severity of seizure was scored using Racine’s scale, and frequency of seizures was determined by 2 individuals using double-blind methods. Mice were sacrificed at the 35th day after SE, and the hippocampus was collected for Western blot analysis.

### Behavioral assay

The general behavioral activities induced by pilocarpine were monitored and recorded through video camera and then analyzed by two researchers blinded to treatment conditions. For the pilocarpine epilepsy models, the behaviors were scored according to Racine’s scale^29^: 0: arrest, wet dog shakes, and normal behavior; 1: facial twitches (nose, lips, and eyes); 2: chewing and head nodding; 3: forelimb clonus; 4: rearing and falling on fore-limbs; 5: imbalance and falling on side or back. Latency was defined as the time after the pilocarpine injection to the first spontaneous seizure onset. Mice who reached stage 4 or 5 were recruited in later experiments.

### Western blot and immunoprecipitation

The cultural neurons and tissue samples from mice infected with or without lentiviral were collected for Western blot analysis. Postsynaptic densities (PSD) were isolated as previously described^31^,^32^. Briefly, neurons and tissue samples were homogenized in homogenization buffer containing 320 mM sucrose, 4 mM HEPES (pH 7.4) and a protease inhibitor cocktail (Roche, Germany) and centrifuged at 1,000 g at 4 °C for 10 min to remove nuclear and cellular debris. The supernatant was centrifuged at 10,000 g at 4 °C for 15 min, and the pellet was lysed by hypoosmotic shock in 4 mM HEPES (pH 7.4) and centrifuged at 25,000 g at 4 °C for 20 min. The resulting pellet was resuspended in 50 mM HEPES (pH 7.4), 2 mM EDTA and 0.5% Triton X-100, and the solution was rotated at 4 °C for 15 min and centrifuged at 32,000 g at 4 °C for 20 min to obtain the pellet. The pellet was resuspended in the same buffer containing 0.5% Triton X-100 and centrifuged at 200,000 g at 4 °C for 20 min. The pellet was resuspended in 50 mM HEPES (pH 7.4) and 2 mM EDTA and collected as the PSD fraction.

Protein concentrations were measured using a BCA assay (Dingguo, Beijing, China). Protein samples (20–50 μg, depending on which protein was going to be detected) were separated on a 12% SDS-PAGE gel and electrotransferred onto a 0.45 μm PVDF membrane (Millipore, Billerica, MA, USA). The membranes were blocked with 5% nonfat dry milk in TBST for 1 h at room temperature (RT) and incubated with rabbit anti-NR4A1 (Proteintech, Wuhan, China), anti-NR2B (Proteintech), anti-NR2A (Proteintech), anti-PSD-95 (Proteintech), anti-phosphor-NR2B (p-NR2B, Tyr1472, Cell Signaling Technology) and mouse anti-GAPDH (Proteintech) antibodies overnight at 4 °C. The blots were washed and incubated for 1 h with HRP-conjugated anti-rabbit or anti-mouse secondary antibodies (Proteintech). The blots were washed again in TBST, and the bands were visualized using an ECL reagent (Thermo, Marina, CA, USA) and a Fusion FX5 image analysis system (Vilber Lourmat, Marne-la-Vallée, France). Relative protein expression levels were calculated using Quantity One software (Bio-Rad, CA, USA) with normalization to the GAPDH signal. For coimmunoprecipitation, protein extracts (approximately 100 mg) from mouse hippocampal tissues were homogenized and mixed with IP lysis buffer. Equal amounts of the proteins were incubated with 2 μl of rabbit IgG (Abcam, Cambridge, UK) as a polyclonal-isotype control or 4 μl NR4A1 or 4 μl NR2B antibodies (Proteintech) overnight at 4 °C followed by incubation with 20 μl protein A/G agarose beads (Santa Cruz Biotechnology) for 2 h at 4 °C. The protein-bead complex was then washed five times and collected by centrifugation, and the samples were mixed with 2 × loading buffer and heated at 95 °C for 5 min. The samples were subjected to Western blotting with same set of antibodies as above.

### Triple-label immunofluorescence and confocal microscopy

Immunofluorescence was performed as previously described in our laboratory^25^,^26^. In brief, the tissue sections were deparaffinized, rehydrated and underwent antigen recovery. Then, the sections were permeabilized with 0.4% Triton X-100 for 10 min and blocked using normal goat serum (Zhongshan Golden Bridge Inc., Beijing, China) for 1 h to eliminate nonspecific staining and incubated in a mixture of rabbit anti-NR4A1 antibody (Proteintech), mouse anti-microtubule-associated protein 2 (MAP2) antibody (Zhongshan Golden Bridge) and chicken anti-astrocyte marker glial fibrillary acidic protein (GFAP, Zhongshan Golden Bridge) antibody or goat anti-Aldehyde Dehydrogenase 1 Family Member L1 (Aldh1L1) antibody (Santa Cruz) overnight at 4 °C. Cells were washed using PBS and incubated with Alexa Fluor-350 goat anti-mouse IgG, Alexa Fluor-488 goat anti-rabbit IgG, and Alexa Fluor-594 goat anti-chicken IgG or Alexa Fluor-594 donkey anti-goat IgG (Zhongshan Golden Bridge) in the dark for 2 h at 37 °C. Cells were washed again in PBS, mounted, sealed, and dried overnight. Finally, the images were captured using confocal laser scanning microscopy (Leica, Wetzlar, Germany).

### Statistical analysis

All data are shown as the mean ± standard deviation (SD) of three independent experiments and were analyzed using Student’s t-test and one-way ANOVA to determine the significance. P values less than 0.05 were considered statistically significant.

## Results

### Clinical characteristics

There were no significant differences in age and gender between the epilepsy patients and controls (*P* > 0.05). In current study, the mean age of the epilepsy patients was 25.96 ± 11.04 (range 8–50 years) with 13 males and 11 females, and the mean time from the onset of seizures was 10.67 ± 7.41 years (range 3–35 years) (Table 1). All of the patients had taken three or more antiepileptic drugs (AEDs) and had recurrent seizures for at least 3 years. The control group had a mean age of 24.10 ± 13.23 years (range 8–48 years) and consisted of 5 men and 5 women (Table 1).

### NR4A1 expression in TLE patients

**Figure 1:**
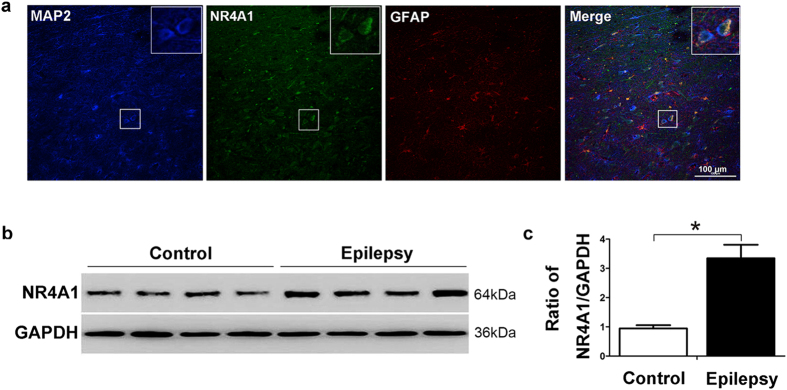
NR4A1 expression in TLE patients. (**a**) Immunofluorescence labeling of NR4A1 in the human neocortex. NR4A1 (green) and MAP2 (blue) (not GFAP, red) are co-expressed in the neocortex of TLE patients. The scale bar = 100 μm. (original magnification ×200). (**b**) The representative Western blot shows NR4A1 expression in the neocortex of control patients (n = 10) or TLE patients (n = 24). (**c**) Three such experiments were quantified from (**b**) by measuring the intensity of the NR4A1 proteins relative to the GAPDH control. (*P < 0.05, compared to control group). The bars indicated the mean ± SD.

First, immunofluorescence was used to determine the distribution of NR4A1 in the brain. NR4A1 immunofluorescence staining was predominantly observed in the cytomembrane and cytoplasm in human brain tissues (Fig. 1a). The neuron marker MAP2 (blue) and NR4A1 (green) were co-expressed in neurons but not with glial fibrillary acidic protein (GFAP, a marker of astrocytes, red). Furthermore, we examined the distribution of NR4A1 in primary hippocampal neurons, and the results showed that NR4A1 was distributed in the cytoplasm and dendrites of neurons (Supplementary Figure 1).

NR4A1 protein levels in the neocortex of TLE patients and control individuals were detected by Western blotting. As shown in Fig. 1b, compared with the controls, NR4A1 expression was elevated in patients with TLE. The expression of NR4A1 was normalized by calculating the intensity ratio of the bands according to the corresponding levels of GAPDH. The analysis showed that the NR4A1 proteins levels dramatically increased 3.39-fold in the neocortex compared with the control group (*P* < 0.05, Fig. 1c).

### NR4A1 expression in a pilocarpine-induced epilepsy mouse model

**Figure 2:**
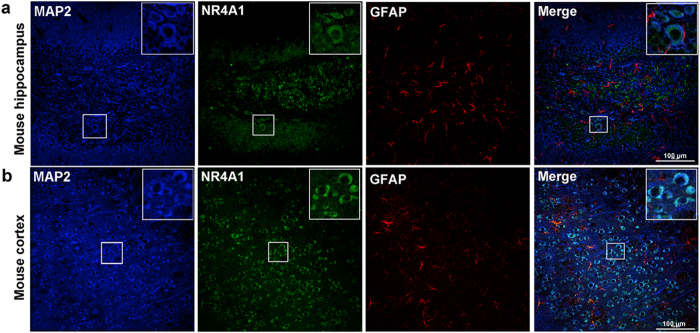
Immunofluorescence labeling of NR4A1 in the mouse models. NR4A1 (green) and MAP2 (blue) (not GFAP, red) are co-expressed in the hippocampus (**a**) and the cortex (**b**) of mice. The scale bar = 100 μm. (original magnification ×200).

NR4A1 immunofluorescence staining was principally observed in the cytomembrane and cytoplasm in the mouse hippocampus (Fig. 2a) and cortex (Fig. 2b). The neuron marker MAP2 (blue) and NR4A1 (green) were co-expressed in neurons, but not with glial fibrillary acidic protein (GFAP, a marker of astrocytes, red). Furthermore, NR4A1 (green) were not co-expressed with Aldehyde Dehydrogenase 1 Family Member L1 (Aldh1L1, a marker of pan-astrocyte, red) (Supplementary Figure 2). It showed that NR4A1 can not distribute in astrocytes, which is consistent with the results from Figs 1a and  2.

**Figure 3:**
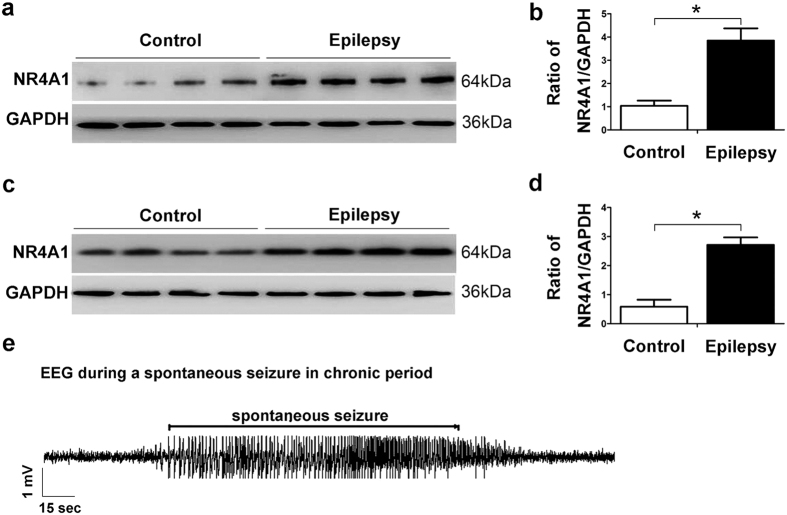
NR4A1 expression in a pilocarpine-induced epilepsy mouse models. The representative Western blot images show NR4A1 expression in the hippocampus (**a**) and the cortex (**c**) of mice with SRS (n = 12) or no SRS (n = 6). (**b, d**) Three such experiments were quantified from (**a, c**) by measuring the intensity of the NR4A1 proteins relative to the GAPDH control (*P < 0.05, compared to no SRS control group). The bars indicated the mean ± SD. (**e)** Representative hippocampal EEG recordings in the pilocarpine model during a spontaneous seizure in the chronic phase of epilepsy.

To extend the results from the analysis of human tissues and exclude the possibility that altered NR4A1 expression may be caused by AEDs in patients with epilepsy, NR4A1 expression levels in the hippocampus and cortex of epileptic and control mice were detected by Western blotting. We observed significantly increased NR4A1 expression in both the hippocampus (3.72-fold, *P* < 0.05, Fig. 3a and 3b) and cortex (4.23-fold, *P* < 0.05, Fig. 3c and 3d) in epileptic mice compared with the controls (n = 6). Our results indicate that NR4A1, a downstream target of the CREB, may be involved in epileptic seizures.

The bursts of spiking activity lasting more than 15s, followed by depressed background activity, could be detected in the SRS groups (considered the epilepsy group) with an EEG recording in chronic periods, but could not be detected in the non-SRS groups (considered the control group). The EEG recording demonstrated that the pilocarpine epileptic mice were characterized by high-frequency and large-amplitude polyspikes (Fig. 3e).

### *In vivo* depletion of NR4A1 by lentivirus attenuates behavioral activities of seizure

**Figure 4:**
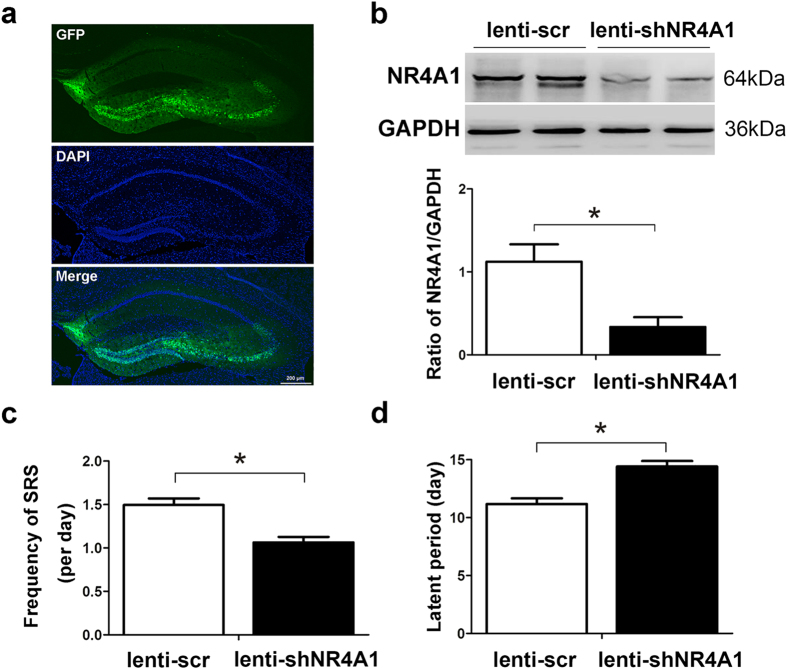
Effect of NR4A1 knockdown on seizure severity. (**a**) Images show GFP expression in dentate gyrus 5 days after lentivirus injection, and cell nuclei were counterstained with DAPI. The scale bar = 200 μm. (**b**) Western blots images show NR4A1 expressions with or without injection of lenti-shNR4A1 at day 5. Statistical analysis showing normalized NR4A1 expression before and after recombinant lentiviral injection. Data indicated the mean ± SD. (*P < 0.05). (**c**) NR4A1 knockdown in epileptic mice inhibits the frequency of seizure. (**d**) Effect of lenti-sh-NR4A1 on the latency of seizures in pilocarpine-induced mouse models. (*P < 0.05, compared to lenti-scr group).

To further investigate the effects of the NR4A1 on seizure severity in animal models, we first assessed whether the lentiviral vector of NR4A1 shRNA (lenti-shNR4A1) was successfully administered in the hippocampus. Mice treated with scramble lentivirus (lenti-scr) infusions at the corresponding time points were used as controls. On the 5th day after injection, lentivirus bearing GFP was successfully localized in the dentate gyrus area (Fig. 4a). Western blot analysis showed that the expression of NR4A1 was significantly decreased in all mice treated with lenti-shNR4A1 compared with the lenti-scr group (*P* < 0.05, Fig. 4b), indicating that the lentiviral vector had been successfully delivered into the hippocampus. To confirm that the reduction in NR4A1 was the cause rather than the consequence of seizure attenuation, we detected the expression of NR4A1 after lentiviral infection in mouse primary hippocampal neurons. Our results showed that the expression of NR4A1 was also markedly decreased in the lenti-shNR4A1 group compared with the lenti-scr group (*P* < 0.05, Supplementary Figure 3).

In behavior analysis of the pilocarpine model of seizure, we found that NR4A1 inhibition (lenti-shNR4A1) significantly inhibited seizure frequency as compared to the lenti-scr groups (*P* < 0.05, Fig. 4c). Because NR4A1 was observed to affect the frequency of seizure, we hypothesized that NR4A1 may also affect seizure latency. We verified this hypothesis by recording the time of onset of the first generalized clonic or tonic seizure. Statistical analysis showed that NR4A1 knockdown significantly increased seizure latency compared to the lenti-scr groups (*P* < 0.05, Fig. 4d). These data suggest that down-regulation of NR4A1 decreased seizure susceptibility.

### Lenti-shNR4A1 down-regulates surface expression of NR2B in the hippocampus of mouse models

**Figure 5:**
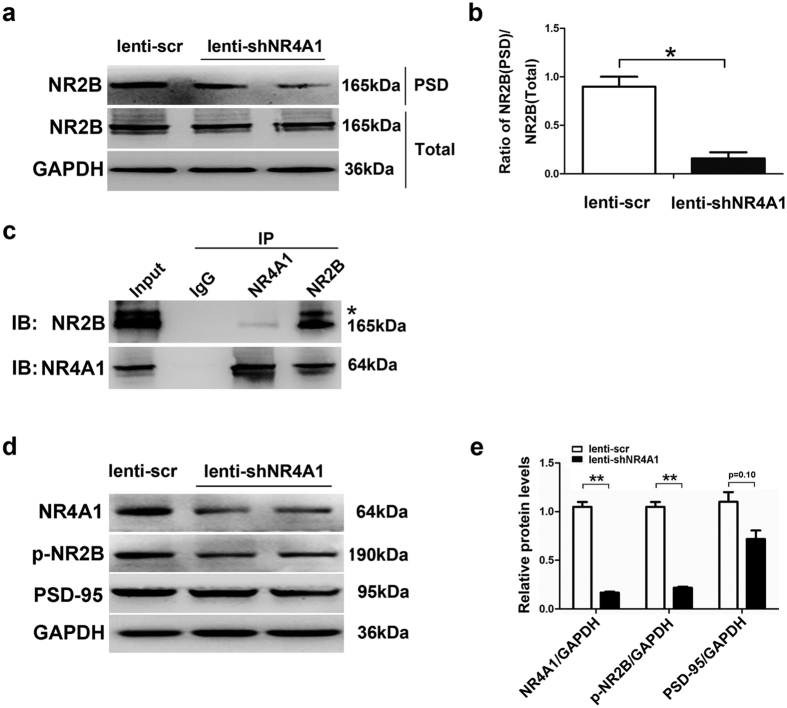
Lenti-shNR4A1 down-regulates the expression of NR2B in the postsynaptic density. The postsynaptic density (PSD) was prepared from epileptic mice infected with lentiviral through ultra-centrifugation. (**a**) Sample Western blots showing PSD and total levels of NR2B in the lenti-scr and lenti-sh-NR4A1 groups in pilocarpine-induced seizure mice. (**b**) Three such experiments were quantified from (**a**) by measuring the intensity of PSD/total proteins (*P < 0.05, compared to lenti-scr group). (**c**) Coimmunoprecipitation was used to assess the interaction between NR4A1 and NR2B. The results indicated that reciprocal coimmunoprecipitation of these two proteins by anti-NR4A1 or anti-NR2B antibodies, demonstrating the interaction of NR4A1 and NR2B. *, Non-specific band. (**d**) Sample Western blots showing the expression of p-NR2B (Tyr1472) and PSD-95 in the lenti-scr and lenti-shNR4A1 groups in epileptic mice. (**e**) Three such experiments were quantified from (**d**) by measuring the intensity of the p-NR2B or PSD-95 proteins relative to the GAPDH control. (**P < 0.01, compared to lenti-scr group). The bars indicated the mean ± SD.

As a CREB target gene, NR4A1 can be induced by NMDAR activation in the cerebral cortex^33^. NR2B overexpression increases NR4A1 expression^33^. To determine whether NR4A1 altered NR2B trafficking, we compared NR2B expression in total and postsynaptic densities (PSD) by Western blot. A significant reduction in NR2B expression in the PSD was observed in the lenti-shNR4A1 group compared with the lenti-scr group, whereas the total NR2B subunit expression was unchanged (Fig. 5a and b). The increase in the PSD but not total NR2B suggests that NR4A1 likely influences NR2B trafficking to the surface or NR2B stability on the plasma membrane. Next, we infected primary hippocampal neurons with lenti-shNR4A1 or lenti-scr for 72 h, and the postsynaptic density (PSD) was also prepared through ultra-centrifugation. The results showed that NR2B in the PSD was significantly reduced in the lenti-shNR4A1 group compared with the lenti-scr group (*P* < 0.01, Supplementary Figure 4). Interestingly, the surface expression of NR2A was not observably changed (Supplementary Figure 5), although the molecular mechanism underlying is not yet clear.

A direct interaction could strongly alter NR2B surface trafficking^34^. Thus, we tested the binding between NR4A1 and NR2B *in vivo* by coimmunoprecipitation experiments using brain lysates. The results indicated coimmunoprecipitation of NR4A1 and NR2B by anti-NR4A1 antibodies (Fig. 5c), demonstrating the interaction of NR4A1 and NR2B, and this interaction was validated by reciprocal coimmunoprecipitation of these two proteins by anti-NR2B antibodies (Fig. 5c). To further elucidate how NR4A1 inhibition affects surface NR2B, we detected the expression of phosphor-NR2B (Tyr1472, p-NR2B) and PSD-95 in epileptic mice infected with lentivirus by Western blot analysis. The results showed that the p-NR2B level was significantly decreased in the lenti-shNR4A1 group compared with the lenti-scr group (*P* < 0.05, Fig. 5d and 5e), indicating the enhancement of NR2B internalization. Although the expression of PSD-95 was reduced in the lenti-shNR4A1 group compared with the lenti-scr group, it was not statistically significant (*P* = 0.10, Fig. 5d and 5e), suggesting that NR4A1 inhibition did not affect the NR2B-mediated internalization. Taken together, our results showed that NR4A1 knockdown could partly decrease surface NR2B by promoting NR2B internalization.

## Discussion

The principal finding of this study was that NR4A1 plays an important role in modifying seizures. In particular, we found that NR4A1 levels were significantly increased in patients with TLE and epileptic mice. NR4A1 knockdown alleviated seizure severity and prolonged the latency to first spontaneous seizure. Coimmunoprecipitation studies showed that NR4A1 could interact with NR2B. Furthermore, NR4A1 inhibition decreased surface NR2B by enhancing NR2B internalization. Therefore, we proposed that the NR4A1 pathway could play an important role in regulating epileptic seizures and may be involved in the development of refractory epilepsy in humans.

NR4A1, an immediate-early gene, is involved in a wide array of important biological processes, including cell apoptosis, brain development, and glucose metabolism^9^. Previous studies have shown that NR4A1 played a key role in mediating neuronal differentiation during central nervous system (CNS) development, and NR4A1 was significantly induced in the CNS by stimuli such as seizures^10^,^11^. In addition, NR4A1 expression was induced in the hippocampus of mice in an epileptic model^10^,^13^,^14^. Currently, the molecular and cell biological mechanisms by which NR4A1 acts in epilepsy are largely unexplored. Immunofluorescence studies in TLE patients revealed that NR4A1 protein is predominantly expressed in neurons as shown by co-localization with the dendritic marker, MAP2 (Fig. 1a). Furthermore, NR4A1 was distributed in the cytoplasm and dendrites of primary hippocampal neurons (Supplementary Figure 1). The results in experimental mice also showed that NR4A1 protein is largely expressed in neurons of the hippocampus and cortex (Fig. 2). Using Western blotting analysis, we found that the expression of NR4A1 significantly increased in the neocortex of TLE patients compared to non-epileptic controls (Fig. 1). To eliminate the effects of AEDs on these experimental results, we verified the results using a chronic pilocarpine epileptic mouse model. The results showed that NR4A1 expression dramatically increased in the hippocampus and cortex of epileptic mice displaying spontaneous recurrent seizures (SRS) compared with no SRS controls (Fig. 3a–3d). Therefore, these results provide direct evidence that epileptic seizures up-regulate NR4A1 expression.

The pilocarpine-induced mouse model of epilepsy shows specific changes in the selectively vulnerable hippocampal formation, which displays similar characteristics to human TLE. To explore the role of NR4A1 in epilepsy, we inhibited the expression of NR4A1 by lentivirus transfection. We found that NR4A1 knockdown alone did not cause epileptic seizures (data not shown). In addition, the depletion of NR4A1 (lenti-shNR4A1) inhibited the frequency of seizures compared with the lenti-scr groups (Fig. 4c). Furthermore, NR4A1 inhibition significantly increased seizure latency (Fig. 4d). These data suggest that downregulation of NR4A1 decreased seizure susceptibility.

The inhibitory and excitatory neurotransmitters in the brain are important modulators of hyper-excitability in epilepsy patients and experimental animal models^35^. NMDARs are critical regulators of glutamatergic synaptic transmission in the CNS, and their function and trafficking are regulated by multiple physiological and pathological processes^36^. The trafficking of NMDARs may regulate the balance between excitatory and inhibitory postsynaptic receptor numbers, maintain SE^37^ and shape synaptic maturation and plasticity^38^. During epileptic seizures, the increased number of cell surface NMDARs has important implications for cell injury and neuron death, and NMDAR blockade can provide neuroprotection against SE or excitotoxicity^39^,^40^,^41^. To investigate whether NR4A1 plays a role in regulating the surface expression of NR2B, we compared the expression of total and PSD NR2B by Western blot analysis. In the lenti-shNR4A1 group, a significant reduction in NR2B expression in the PSD was observed compared with the lenti-scr group (Fig. 5a and 5b). The increase in the PSD but not total NR2B suggests that NR4A1 likely influences NR2B trafficking to the surface or its stability on the plasma membrane. NR4A1 inhibition may retard NR2B trafficking from the intracellular pool to the surface and also decreases their cell surface expression, which may suppress epilepsy. A direct interaction could strongly alter NR2B surface trafficking^42^. As shown in Fig. 5c, reciprocal coimmunoprecipitation of NR4A1 and NR2B demonstrated the interaction of NR4A1 and NR2B. Under normal conditions, NR2B internalization occurs through the clathrin-mediated pathway and is mediated primarily by an interaction between the YEKL motif contained within the NR2B distal C termini and the clathrin adaptor protein 2 (AP2), which initiates clathrin-dependent internalization^43^. The dephosphorylation of Tyr1472 in the NR2B subunit promotes internalization of NR2B via the clathrin pathway^43^, leading to increased surface NR2B. Moreover, PSD-95 promotes NR2B receptor clustering and its surface expression^43^ and inhibits NR2B-mediated internalization^44^. Our results showed that the expression of p-NR2B (Tyr1472) but not PSD-95 was significantly decreased in the lenti-shNR4A1 group compared with the lenti-scr group (Fig. 5d and 5e), indicating that enhancement of NR2B internalization could lead to decreased surface NR2B. The reduction of NR4A1 could partly decrease surface NR2B by promoting NR2B internalization and then regulate seizure susceptibility. The detailed mechanisms of whether NR4A1 influences NR2B internalization is mediated by an interaction between the YEKL motif and the AP2 complex remains to be illuminated in the future studies.

The data from human brain tissues reported in the present study have some limitations. For ethical reasons and limitations, we could not obtain normal human brain tissues; thus, we used histologically normal brain tissues obtained from temporal lobectomy performed to treat head trauma^25^,^26^. Meanwhile, the brain tissue samples of the neocortex with TLE could only be obtained from drug-resistant epileptic patients. For this reason, a pilocarpine-induced epileptic mouse model that has been broadly used as a model for human TLE has obvious advantages in repeating the results of the epileptic patients^28^,^45^,^46^. Thus, we performed our study on brain tissues of both TLE patients and experimental mice using two complementary methods to further investigate the expression of NR4A1 and the potential mechanism of TLE. Our results from the epileptic mouse model also provide direct evidence that seizure activities alter NR4A1 expression levels in the hippocampus and cortex (Figs 1, 2, 3).

The present study provided direct evidence that NR4A1 is up-regulated in patients with epilepsy and in mouse models and that NR4A1 knockdown alleviates seizure attacks, which may lead to a novel intervention strategy for human epilepsy. Coimmunoprecipitation studies showed that NR4A1 could interact with NR2B. Furthermore, NR4A1 knockdown could decrease surface NR2B by enhancing NR2B internalization. However, the detailed mechanisms of how NR4A1 influences NR2B internalization remain to be elucidated in the future.

## Additional Information

**How to cite this article**: Zhang, Y. *et al*. NR4A1 Knockdown Suppresses Seizure Activity by Regulating Surface Expression of NR2B. *Sci. Rep.* **6**, 37713; doi: 10.1038/srep37713 (2016).

**Publisher's note:** Springer Nature remains neutral with regard to jurisdictional claims in published maps and institutional affiliations.

## Supplementary Material

Supplementary Information

## Figures and Tables

**Table 1:** Clinical characteristics of intractable epilepsy and control patients.

Patient	Sex (M/F)	Age (years)	Course (years)	AEDs before surgery	Source of organization	Pathologic results
1	F	18	6	LTG/OXC/PB/VPA	RTN	G
2	M	22	18	PB/CBZ/VPA/OXC	LTN	NL/G
3	M	36	15	CBZ/PHT/LTG	LTN	NL/G
4	F	14	8	VPA/CBZ/LTG/PB	RTN	NL/G
5	M	14	4	TPM/VPA/CBZ	LTN	G
6	M	27	10	VPA/PB/LEV/OXC	RTN	G
7	F	33	19	OXC/CBZ/TPM/PB	RTN	NL/G
8	F	50	35	CBZ/VPA/PHT	LTN	G
9	M	45	10	VPA/TPM/PHT	LTN	G
10	F	40	24	CBZ/TPM/LTG	RTN	NL/G
11	F	19	10	VPA/CBZ/PB/OXC	LTN	NL/G
12	M	18	8	VPA/OXC/PB	RTN	G
13	M	11	3	VPA/CBZ/TPM/PB	LTN	G
14	F	26	10	CBZ/PB/LEV	RTN	NL/G
15	F	8	3	CBZ/PB/LTG/OXC	RTN	NL/G
16	F	22	3	CBZ/PHT/OXC	LTN	G
17	M	29	8	VPA/CBZ/PHT	RTN	G
18	M	15	6	CBZ/LTG/LEV	RTN	NL/G
19	M	37	3	CBZ/PHT/VPA	LTN	G
20	F	39	12	PHT/CBZ/VPA	LTN	NL/G
21	F	30	11	VPA/PB/PHT	LTN	G
22	M	24	10	CBZ/PB/LEV/PHT	RTN	NL/G
23	F	26	11	CBZ/LTG/LEV/VPA	RTN	NL/G
24	F	20	9	CBZ/PB/LEV	RTN	NL/G
**Patient**	**Sex (M/F)**	**Age (years)**	**Disease diagnosis**	**Source of organization**	**Pathologic result**	
1	F	20	Brain trauma	RTN	Normal	
2	M	12	Brain trauma	LTN	Normal	
3	F	16	Brain trauma	RTN	Normal	
4	M	21	Brain trauma	LTN	Normal	
5	F	40	Brain trauma	RTN	Normal	
6	M	38	Brain trauma	LTN	Normal	
7	F	8	Brain trauma	LTN	Normal	
8	F	48	Brain trauma	LTN	Normal	
9	M	21	Brain trauma	RTN	Normal	
10	M	17	Brain trauma	RTN	Normal	

F: female, M: male, AEDs: antiepileptic drugs, LTG: lamotrigine, TPM: topiramate, CBZ: carbamazepine, PHT: phenytoin, PB: phenobarbital, VPA: valproic acid, LEV: levetiracetam, OXC: oxcarbazepine, RTN: right temporal neocortex, LTN: left temporal neocortex, NL: neuronal necrosis, G: gliosis.
