# Corrigendum: NR4A1 Knockdown Suppresses Seizure Activity by Regulating Surface Expression of NR2B

**DOI:** 10.1038/srep42935

**Published:** 2017-02-24

**Authors:** Yanke Zhang, Guojun Chen, Baobing Gao, Yunlin Li, Shuli Liang, Xiaofei Wang, Xuefeng Wang, Binglin Zhu

Scientific Reports 6: Article number: 3771310.1038/srep37713; published online: 11 23 2016; updated: 02 24 2017

This Article contains errors in the Experimental procedures section under subheading ‘Lentiviral vector injections’.

“We recorded 24 h/day for 21 consecutive days, beginning on the 14th day after SE”.

should read:

“We recorded 24 h/day for 21 consecutive days, beginning on the 4th day after SE”.

