# Supplementary material for: NR4A1 Knockdown Suppresses Seizure Activity by Regulating Surface Expression of NR2B
Source: Sci Rep. 2016 Nov 23;6:37713. doi: 10.1038/srep37713 (PMC5120300; doi:10.1038/srep37713)
Supplement: Supplementary Information [file srep37713-s1.pdf]

Supplementary Information

NR4A1 Knockdown Suppresses Seizure Activity by Regulating Surface Expression of NR2B

Yanke Zhang [1], Guojun Chen [1], Baobing Gao [2], Yunlin Li [3], Shuli Liang [4], Xiaofei Wang [5], Xuefeng Wang [1, 6, *], Binglin Zhu [1, *]

[1] Department of Neurology, The First Affiliated Hospital of Chongqing Medical University, Chongqing Key Laboratory of Neurology, 1 Youyi Road, Chongqing 400016, China

[2] Department of Neurology, Chongqing General Hospital, 104 Pipashan Street, Chongqing 400010, China

[3] Department of Neurosurgery, The Affiliated Children's Hospital of Capital Institute of Pediatrics, 2 Yabao Road, Beijing 100020, China

[4] Department of Neurosurgery, The First Affiliated Hospital of PLA General Hospital, 51 Fucheng Road, Beijing 100048, China

[5] Department of Neurology, The Affiliated Children's Hospital, Capital Medical University, 56 Nanlishi Road, Xicheng District, Beijing 100032, China

[6] Center of Epilepsy, Beijing Institute for Brain Disorders, 10 Xitoutiao, Youanmen, Fengtai District, Beijing 100069, China

* Corresponding author: Xuefeng Wang, Email: xfyp@163.com and Binglin Zhu, Email: zhubinglin0311@163.com, Phone & fax: +862389011834.

Supplementary Methods

Hippocampal neuron cultures and lentivirus infection.

Cultured hippocampal neurons were prepared from the mouse embryos on day 18 of gestation as previously detailed[1]. The brains were removed from the fetuses, and the hippocampi were isolated. The dissected tissue was minced into small pieces and then gently digested with trypsin. Then, the cells were centrifuged, and the pellet was resuspended in Neurobasal medium (Gibco, Invitrogen, USA) supplemented with 2% B27 and 100 units/ml penicillin/streptomycin. Cultures were maintained at 37℃ atmosphere with 5% CO_2 and medium was changed 24 h after plating and every 3 days thereafter. Follow-up experiments were performed after 7 days in culture. Neurons were plated on poly-D-lysine (0.1 mg/ml, Sigma Aldrich)-coated 10 cm dishes at 8×10^6 cells/dish for postsynaptic densities fractions preparation and Western blot or on poly-D-lysine-coated coverslips at 0.5×10^6 cells/coverslip for immunofluorescence. Neurons were infection with lenti-shNR4A1 and lenti-scr for 72 h.

Immunofluorescence and confocal microscopy.

For immunofluorescence, neurons were plated on poly-D-lysine-coated coverslips at 0.5×10^6 cells/coverslip and washed twice with PBS and fixed by 4% (v/v) paraformaldehyde (PFA) for 10 min at room temperature (RT). Then, neurons were permeabilized with 0.4% Triton X-100 for 10 min and blocked using normal goat serum (Zhongshan Golden Bridge) for 1 h to eliminate nonspecific staining and then incubated in a mixture of rabbit anti-NR4A1 antibody (Proteintech), mouse anti-microtubule-associated protein 2 (MAP2) antibody (Zhongshan Golden Bridge) overnight at 4℃. Cells were washed using PBS and incubated with Alexa Fluor-350 goat anti-mouse IgG, Alexa

Fluor-488 goat anti-rabbit IgG in the dark for 2 h at 37℃. 5 μM DRAQ5 (Abcam, ab108410) was added to the secondary antibody mixture to label nuclear DNA. Cells were washed again in PBS, mounted and sealed. Finally, the images were captured using confocal laser scanning microscopy (Leica, Wetzlar, Germany).

Supplementary Figures

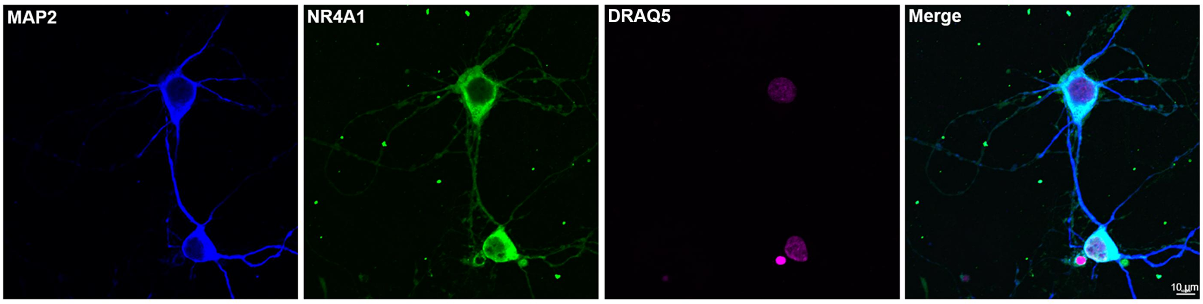

Supplementary Figure 1: Immunofluorescence labeling of NR4A1 in primary hippocampal neurons. Neurons are stained with anti-NR4A1 (green), anti-MAP2 (blue) and DRAQ5 nuclear stain (purple). NR4A1 and MAP2 are co-expressed in the primary hippocampal neurons of mouse. NR4A1 was distributed in the cytoplasm and dendrites of neurons. Scale bar = 10 μm. (original magnification ×800).

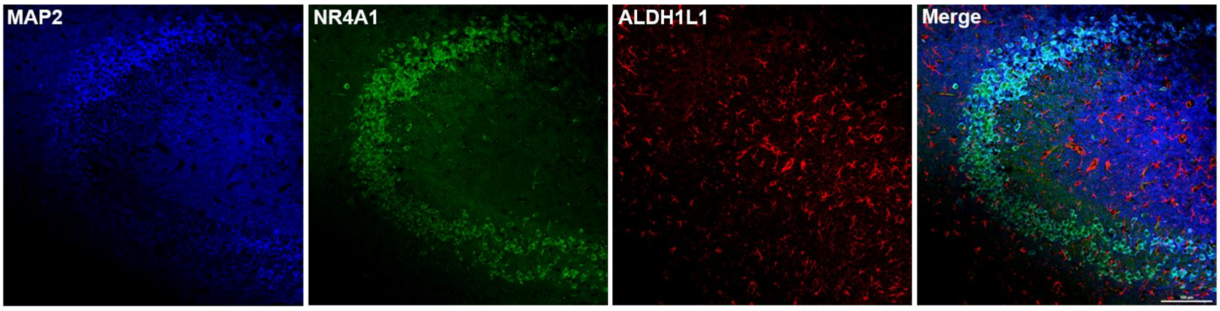

Supplementary Figure 2: NR4A1 did not distribute in astrocyte. Representative confocal images from epileptic mice. Slices are stained with anti-NR4A1 (green), anti-Aldehyde Dehydrogenase 1 Family Member L1 (Aldh1L1, red). NR4A1 was not co-expressed with Aldh1L1 (a marker of pan-astrocyte). Scale bar = 100 μm. (original magnification ×200).

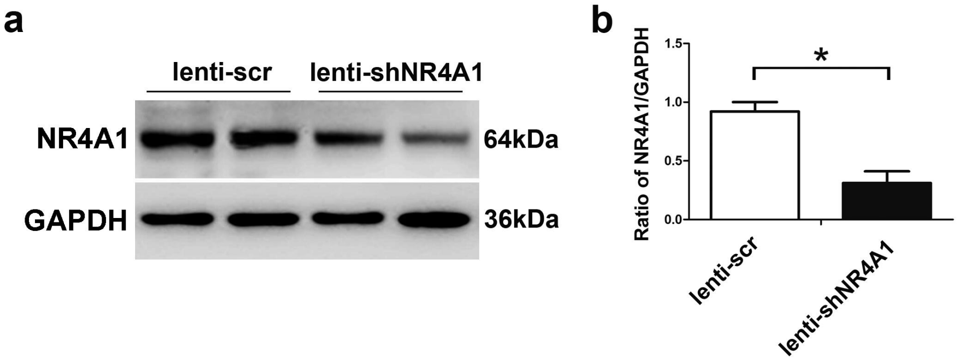

Supplementary Figure 3: Lenti-shNR4A1 down-regulates the expression of NR4A1 in primary

hippocampal neurons. Mouse primary hippocampal neurons were infected with lenti-shNR4A1 or lenti-scr for 72 h. **(a)** Representative Western blot images show NR4A1 expression in primary hippocampal neurons. (**b**) Three such experiments were quantified from (**a**) by measuring the intensity of the NR4A1 proteins relative to the GAPDH control. (* $P < 0.05$, compared to lenti-scr group). The bars indicated the mean±SD.

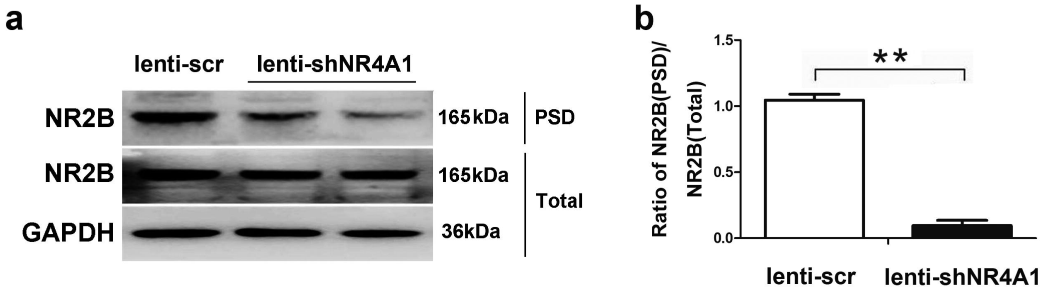

Supplementary Figure 4: Lenti-shNR4A1 down-regulates the expression of NR2B in postsynaptic density. Mouse primary hippocampal neurons were infected with lenti-shNR4A1 or lenti-scr for 72 h, and postsynaptic density (PSD) was prepared through ultra-centrifugation. (**a**) Representative Western blot images show the expression of PSD and total NR2B in the lenti-scr and lenti-sh-NR4A1 group in primary neurons. (**b**) Three such experiments were quantified from (**a**) by measuring the intensity of PSD/total proteins (** $P < 0.01$, compared to lenti-scr group). The bars indicated the mean±SD.

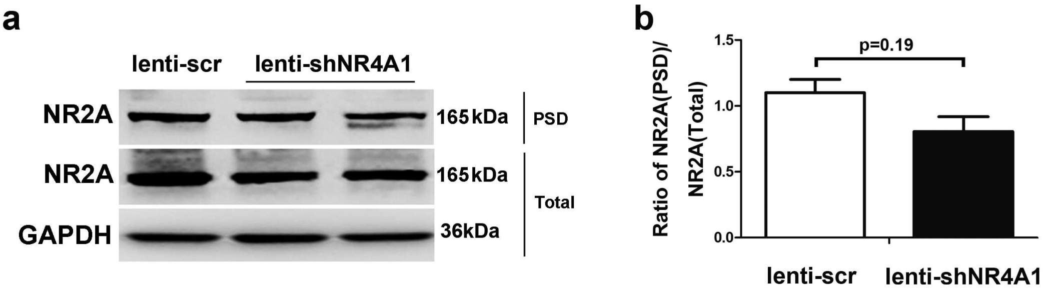

Supplementary Figure 5: Lenti-shNR4A1 did not affect the expression of NR2A in the postsynaptic density. The postsynaptic density (PSD) was prepared from epileptic mice infected with lentiviral through ultra-centrifugation. (a) Sample Western blots showing the PSD and total levels of NR2A in the lenti-scr and lenti-shNR4A1 group in pilocarpine-induced seizure mice. (b) Three such experiments were quantified from (a) by measuring the intensity of PSD/Total proteins ($P = 0.19$, compared to the lenti-scr group). The bars indicated the mean±SD.

References

1. Kaech, S. & Banker, G. Culturing hippocampal neurons. *Nature protocols* **1,** 2406-2415, doi: 10.1038/nprot.2006.356 (2006).
